# Retinitis pigmentosa and molar tooth sign caused by novel AHI1 compound heterozygote pathogenic variants

**DOI:** 10.1186/s12920-021-01089-5

**Published:** 2021-10-09

**Authors:** Chunyan Chen, Jiong Gao, Qing Lv, Chen Xu, Yu Xia, Ailian Du

**Affiliations:** 1 Department of Neurology, Tongren Hospital, Shanghai Jiaotong University School of Medicine, Shanghai, 200336 China; 2 WuXiDiagnostice, No. 31 Yiwei Road Waigaoqiao Pilot Free Trade Zone, Shanghai, 200131 China

**Keywords:** Joubert syndrome, *AHI1* gene, Whole exome sequence, 3D structure, Case report

## Abstract

**Background:**

Joubert syndrome (JS) is a group of rare congenital disorders characterized by cerebellar vermis dysplasia, developmental delay, and retina dysfunctions. Herein, we reported a Chinese patient carrying a new variant in the *AHI1* gene with mild JS, and the 3D structure of the affected Jouberin protein was also predicted.

**Case presentation:**

The patient was a 31-year-old male, who presented difficulty at finding toys at the age of 2 years, night blindness from age of 5 years, intention tremor and walking imbalance from 29 years of age. Tubular visual field and retina pigmentation were observed on ophthalmology examinations, as well as molar tooth sign on brain magnetic resonance imaging (MRI). Whole exome sequence revealed two compound heterozygous variants at c.2105C>T (p.T702M) and c.1330A>T (p.I444F) in A*HI1* gene. The latter one was a novel mutation. The 3D protein structure was predicted using I-TASSER and PyMOL, showing structural changes from functional β-sheet and α-helix to non-functional D-loop, respectively.

**Conclusions:**

Mild JS due to novel variants at T702M and I444F in the *AHI1* gene was reported. The 3D-structural changes in Jouberin protein might underlie the pathogenesis of JS.

## Background

Joubert syndrome (JS) [1] is a group of rare congenital disorders characterized by cerebellar vermis dysplasia, developmental delay with hypotonia, ocular problems, and breathing abnormalities [2, 3]. The diagnostic hallmark of JS is a unique cerebellar and brainstem malformation, depicted as the “molar tooth sign” on brain imaging [4]. Neurological signs are present from the neonatal period, and develop to full neurological dysfunctions during childhood. More seriously, the prognosis of JS is usually disappointing. Dempsey et al. have studied 565 JS patients and found that the case fatality in JS was 7.1% (40/565). The mean age of death was 7.2 years old. Of those who died, the younger children tended to die from respiratory failure and the older from renal failure [5]. But, there are mild JS phenotypes presented with non-syndromic retinitis pigmentosa without neurological symptoms [6], or with mild neurological symptoms and typical molar tooth sign.

To date, 35 genes have been identified as pathogenic for JS [7]. *Abelson helper integration site 1* (*AHI1*) is one of the most common pathogenic gene, which accounts for 7–10% JS population [7, 8]. It encodes the Jouberin protein that functions as a scaffold, which facilitates the formation and function of primary cilia [9]. The mutations in *AHI1* causing JS are supposed due to the disruption of cilium formation and function in ciliated cells of diverse tissues. Herein, we reported a Chinese adult patient, who presented with night blindness and mild walking imbalance but normal mental development. Whole exome sequence (WES) revealed two compound heterozygous variants at NM_017651.4: c.2105C>T (p.T702M) and c.1330A>T (p.I444F) in *AHI1* gene, with the later I444F as a novel mutation. Both variants were predicted to alter the three-dimensional (3D) structure of Jouberin, which might disturb the normal function of this scaffolding protein.

## Case presentation

### Clinical features

The proband was a 31-year-old Chinese Han male patient who was admitted with the chief complaint of “poor vision for 29 years, walking imbalance for 2 years.” He was born to normal non-consanguineous parents and his parents denied any family history of genetic disorders. His critical developing points are as follow: head raising at 3 months, sitting at 6 months, standing at 14 months, and walking at 24 months. At the age of 2 years, he was demonstrated difficult to track and find toys. At the age of 5 years old, he was diagnosed with macular degeneration because of night blindness and findings of fundoscopy at an ophthalmic clinic. Concurrently, his naked eye vision was 0.1 OD and 0.09 OS, and the corrected visual acuity was about 0.5 in both eyes. Subsequently, he was treated with vitamin B12, B1, and B6 during the following 24 years; his visual acuity remained stable but the visual field continued to become narrower. He also experienced intention tremors and walking imbalance in both extremities from the age of 29 years. He couldn’t ride a bicycle because of the narrowed visual field but he can walk down the stairs without holding rails. There were no other symptoms of sensory neuropathy and vestibular dysfunction. Nonetheless, he exhibited normal intelligence and is currently working as a bank staff. He had no Polydactyly.

Physical examination on admission: The patient was conscious, fluent in language, normal in intelligence, tubular visual field, decreased smooth pursuit, and slight nystagmus on bilateral gaze. Although the muscle strength and tension were normal, he has slight intention tremor on finger—nose pointing, unstable at toe -heel walking and straight walking. Minimal-Mental Status Examination (MMSE) score was 30/30, and Montreal Cognitive Assessment (MoCA) score was 29/30 (which was normal). Blood tests, including complete blood count, liver and kidney function, electrolytes, creatine kinase, and lactic acid, were normal. Other examinations, including electrocardiogram, electroencephalogram, abdominal and kidney ultrasound, were normal.

**Fig. 1:**
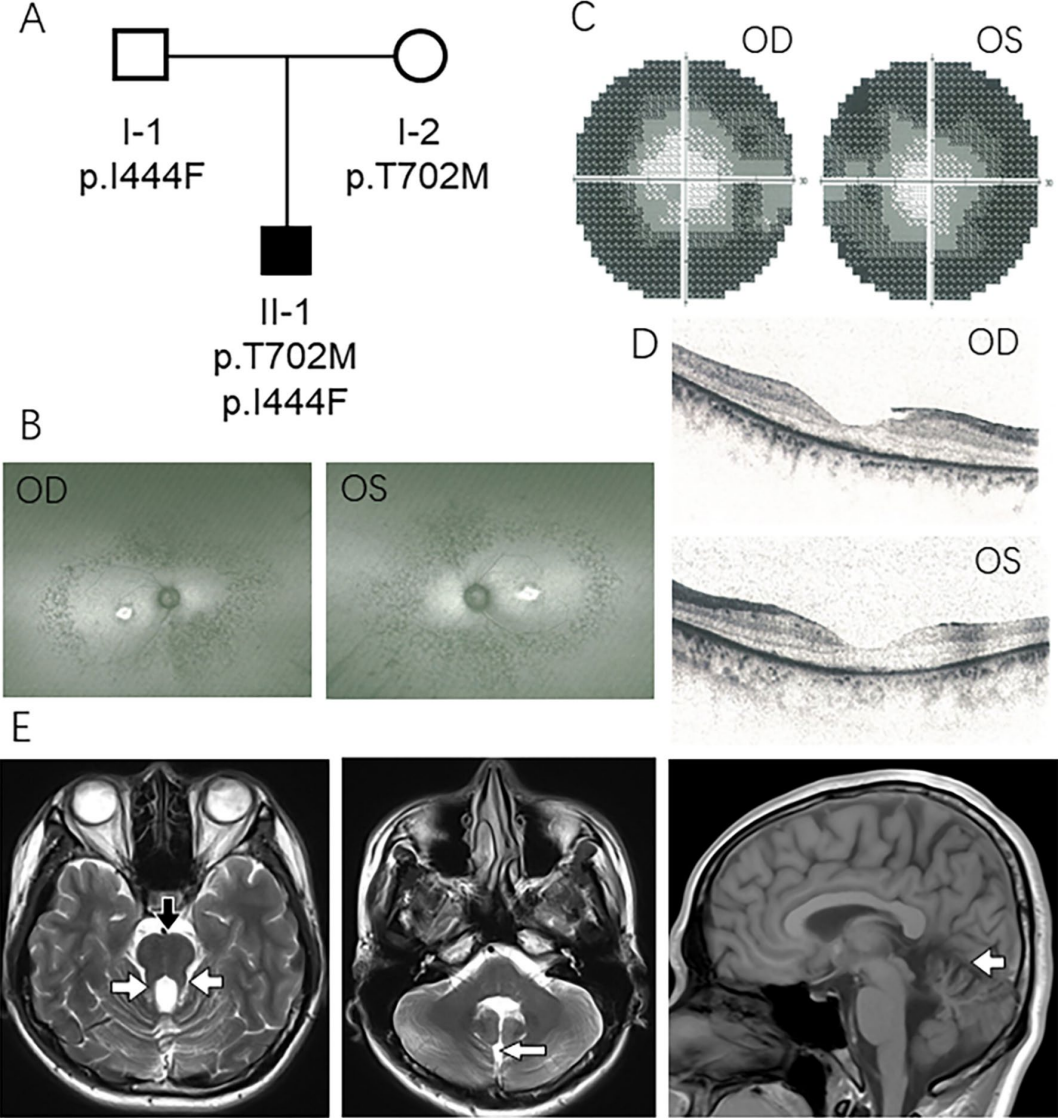
Pedigree of the patient showed that p.T702M and p.I444F mutations were inherited from his mother and father respectively (**A**). Ophthalmic images showed a retina pigmentation on Fundus photograph (**B**), tubular visual field of both eyes (**C**), retinal nerve fiber layer thinning on optical coherence tomography (OCT) images (**D**). Brain T2 MRI axial scan showed that deepened interpeduncular fossa was not obvious (thick arrow), but molar tooth sign (E-1 open arrow), cauda cerebelli hypoplasia, and midline cleft (E-2 white arrow) were clear. Brain T1 MRI sagittal scan showed hypoplasia of superior cerebellar peduncle (E-3 open arrow)

Ophthalmology examinations showed tubular visual field, retina pigmentation on Fundus photograph, retinal nerve fiber layer thinning on optical coherence tomography (OCT) (Fig. 1A–D), which were signs of retinal dystrophy. Brain magnetic resonance imaging (MRI) showed a molar tooth sign (MTS) in the midbrain and cauda cerebelli hypoplasia (Fig. 1E). Based on the clinical characteristics of poor vision, night blindness, tubular visual field, intention tremor and imbalance, combined with retina pigmentation and molar tooth sign on brain MRI, the proband was clinically diagnosed as JS.

### Molecular analyses

**Fig. 2:**
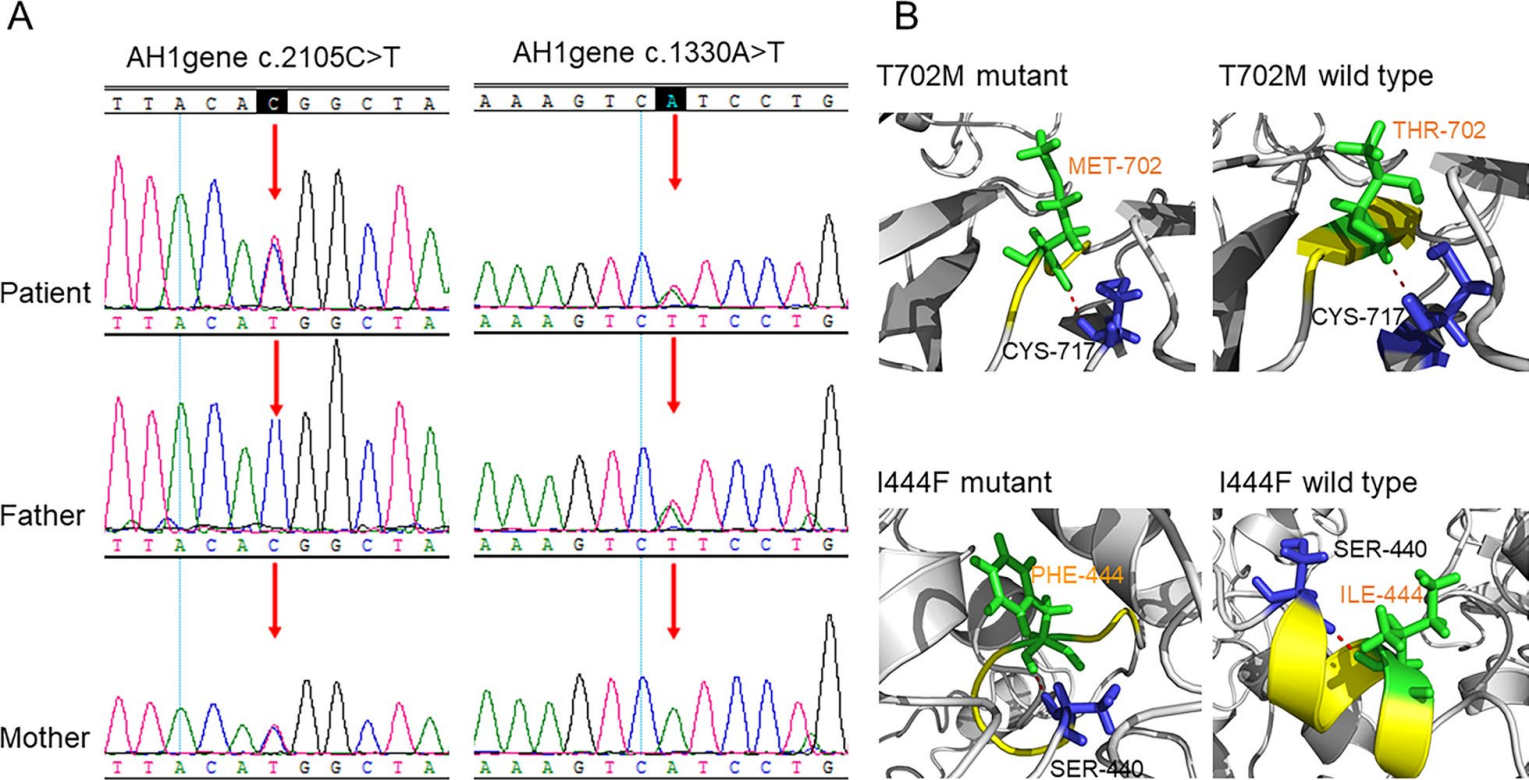
Whole exome sequence results showed heterozygous variants c.2105C>T (p.T702M) and c.1330A>T (p.I444F) in the *AHI1* gene. The c.2105C>T variant is from the mother, and the c.1330A>T variant is from the father (**A**). The 3D structural prediction showed that the T702M mutation changes the structure of Jouberin protein from β-sheet to D-loop, while the I444F mutation alters the structure from α-helix to D-loop (**B**). Both of the mutations alter the conformation of this scaffolding protein and affect the protein function

Total genomic DNA was extracted from the blood of proband and his parents using a DNEasy Blood and Tissue Kit (Qiagen, Hilden, Germany). The genomic DNA of the proband was enriched for coding exons using Agilent SureSelect Exon capture Kit and sequenced on Illumina HiSeq X Ten platform. The WES data were analyzed for single-nucleotide variants and insertion–deletion polymorphisms using Genome Analysis Toolkit (GATK). The pathogenicity of the detected mutations was analyzed using Mutation Taster (http://www.mutationtaster.org/), SIFT (http://sift.jcvi.org/) and PolyPhen-2 (http://genetics.bwh.harvard. edu/pph2/). The results revealed two compound heterozygous variants at NM_017651.4: c.2105C>T (p.T702M) (Fig. 2A) and c.1330A>T (p.I444F) (Fig. 2A) in the *AHI1* gene. The c.2105C>T variant was inherited from the unaffected mother, and the c.1330A>T variant was inherited from the unaffected father (Fig. 1A). According to the American College of Medical Genetics and Genomics (ACMG) guidelines [10], both the c.2105C>T and the c.1330A>T mutations were predicted to be of uncertain significance. However, c.2105C>T has been previously reported as pathogenic (rs756276537) in the compound heterozygous pattern with c.903_910insAp.T304fs*309 [11] or alongside another pathogenic variant (p.E281*) [12]. On the other hand, c.1330A>T mutation has not been reported in the popular public databases, but its pathogenicity could be predicted by Mutation Taster, SIFT and PolyPhen-2. The CADD phred score was 28.2.

### Protein structure prediction

3D structure homology modeling of Jouberin protein encoded by the wild-type *AHI1* gene (NM_017651.4) and mutated *AHI1* gene was predicted using I-TASSER (https://zhanglab.ccmb.med.umich.edu/I-TASSER-MR/), and the architecture was visualized using PyMOL (PyMOL Molecular Graphics System, Version 1.5, Schrödinger, LLC). Homozygous mutation of c.2105C>T (p.T702M) could cause the replacement of a hydroxylic polarly amino acid threonine by a sulfuric non-polarly methionine. The difference of biochemical property of the amino acids would influence protein-folding, which resulted in local 3D structure from β-sheet to D-loop (Fig. 2B), and also decreased the protein stability (ΔΔG of − 0.82 kcal/mol) based on the result of I-Mutant (http://gpcr2.biocomp.unibo.it/cgi/predictors/I-Mutant3.0/I-Mutant3.0.cgi). For the mutation c.1330A>T (p.I444F), even amino acid Ile and Phe are both nonpolar and neutral charged, they still show difference fragment structure with aliphatic and aromatic, respectively. When insight into protein structure, it would change the 3D structure from α-helix to D-loop (Fig. 2B), and decreases protein stability (ΔΔG of − 1.08 kcal/mol).

## Discussion and conclusions

The incidence of JS and related diseases is estimated to be between 1/80,000 and 1/100,000 in live births [1, 13]. Depending on the organ involvement, JS was divided into six subgroups: (1) pure Joubert syndrome (JS), (2) Joubert syndrome with ocular defects (JS-O), Joubert syndrome with renal defects (JS-R), Joubert syndrome with oculorenal defects (JS-OR), Joubert Syndrome with Hepatic Defects (JS-H), and Joubert Syndrome with digital-facial-defect (JS-OFD) [14]. The patient in this study exhibited night blindness poor vision at the age of 2 years, intention tremor, and walking imbalance at the age of 29 years. Physical examination showed poor visual acuity, tubular visual field, decreased smooth pursuit, and unstable straight walking. Based on the mild neurological symptoms and typical molar tooth sign on brain MRI, the patient was diagnosed as mild JS with ocular defects (JS-O). The diagnosis was confirmed by the compound heterozygous variants at c.2105C>T (p.T702M) and c.1330A>T (p.I444F) in the *AHI1* gene. Although the mean age of death due to JS was reported as 7.2 years; while this patient survived into the 3rd decade of life. He maintained mild walking imbalance and absence of developmental delay or altered respiratory pattern. He was intellectually normal and continued working in his 3^rd^ decade. The patient is currently treated with regular dose of vitamin B12 1500 μg/day vitamin B1 30 mg/day, vitamin B6 30 mg/day. The patient is satisfied with the current status of his health.

The *AHI1* is a common disease-causing gene in JS population, especially in JS with retinal involvement or central nervous system abnormalities or both [13]. The Jouberin protein encoded by the human *AHI1* gene contains an N-terminal coiled-coil domain, seven WD40 repeats, an SH3 binding domain in the C terminal, suggesting that Jouberin could also function as a scaffolding protein [15]. The most pathogenic mutations in the *AHI1* gene related to JS are nonsense or frameshift mutations, which cause Jouberin protein truncation and loss of function. In addition, rare missense mutations in exons or introns might alter the protein structure or interaction [8]. Valente et al. (2006) have studied 137 JS related disorder families and found no correlations between the phenotype and the type of variant in the *AHI1* gene. Recently, Nguyen et al. (2017) reported four missense mutations in the WD40 domain, causing non-syndromic retinitis pigmentosa with late-onset and mild progression [6]. They analyzed 11 missense mutations in the *AHI1* gene using 3D structure homology modeling and found that missense mutations causing a severe disruptive effect on WD40 structure might be related to severe phenotypes.

In the present case, we identified two missense variants at c.2105C>T (p.T702M) and c.1330A>T (p.I444F) in *AHI1* gene. The T702M variant belongs to the WD40 domain. This variant has been previously reported as disease-causing in a compound heterozygous pattern with the *AHI1* gene c.903_910insAp.T304fs*309 insertion. The clinical features included hypotonia, developmental delay, and respiratory abnormalities, which are the clinical presentations of JS but lacking ocular symptoms [12]. Another report has described a heterozygous pathogenic pattern with *AHI1* gene p.E281*, which exhibited retinal features in addition to pure JS symptoms [12]. However, the impact of T702M variant on protein structure and function has not been clarified. The I444F variant was a de novo mutation located in the area without any specific domain or matrix. Here, we did the 3D structure prediction and showed that these two variants would alter the conformation of the Jouberin protein, by changing β-sheet to D-loop and α-helix to D-loop respectively. These conformational changes might affect protein localization and reduce the protein stability based on in silico stability prediction. Also, Tuz et al. [16] has found direct evidence on V443D mutation adjacent to I444F. They showed that V443D variant altered the Jouberin protein localization and the protein–protein interactions in V443D-transfected cells and animal models.

In conclusion, we reported two missense variants at p.T702M and p.I444F in the *AHI1* gene causing retinitis pigmentosa and molar tooth sign, with the latter one as a novel variant. These two variants would alter the conformation and stability of Jouberin protein, which might affect the localization and stability of the protein. Thus, this report would contribute to the clinical entity and genotype–phenotype correlation between *AHI1* and Joubert syndrome.

## Abbreviations

JS: Joubert syndrome
MRI: Magnetic resonance imaging
*AHI1*: *Abelson helper integration site 1*
WES: Whole exome sequence
MMSE: Minimal-Mental Status Examination
MoCA: Montreal Cognitive Assessment
OCT: Optical coherence tomography
MTS: Molar tooth sign
ACMG: American College of Medical Genetics and Genomics

## Declarations

### Availability of data and materials

The data that support the findings of this study have been deposited into CNGB Sequence Archive (CNSA) [17] of China National GeneBank DataBase (CNGBdb) [18] (https://db.cngb.org/cnsa/) with accession number CNP0002094.
